# Teaching reform and practical exploration of the fundamental nursing skills course in higher vocational nursing education: a quasi-experimental study

**DOI:** 10.3389/fmed.2026.1860199

**Published:** 2026-06-30

**Authors:** Huanli Luo, Huanling Luo, Rongfang Zhang

**Affiliations:** 1 Department of Nursing, Henan Vocational College of Nursing, Anyang, Henan, China; 2 Department of International Education, Henan Vocational College of Nursing, Anyang, Henan, China

**Keywords:** competency-based education, fundamental nursing skills, higher vocational nursing education, simulation-based learning, teaching reform

## Abstract

**Background:**

The transformation of healthcare services and the increasing demand for clinically competent nurses have posed new challenges for higher vocational nursing education. The course Fundamental Nursing Skills plays a pivotal role in cultivating students’ practical abilities; however, traditional teaching approaches often fail to effectively integrate theory with practice.

**Objective:**

This study aimed to evaluate the effectiveness of a competency-based teaching reform implemented in the Fundamental Nursing Skills course within a higher vocational nursing program.

**Methods:**

A quasi-experimental design was adopted. Seventy-five nursing students were assigned to either an experimental group (reformed teaching model, *n* = 38) or a control group (traditional teaching, *n* = 37). The intervention lasted one academic semester. Outcome measures included theoretical knowledge scores, practical skills assessment, learning engagement, and student satisfaction. Data were analyzed using independent-samples t tests and chi-square tests.

**Results:**

The experimental group achieved significantly higher theoretical knowledge scores (82.5 ± 6.4 vs. 76.2 ± 7.0, *t* = 4.26, *p* < 0.001) and practical skills scores (88.6 ± 5.4 vs. 80.7 ± 6.2, *t* = 6.01, p < 0.001) compared with the control group. Learning engagement and overall satisfaction were also significantly higher in the experimental group.

**Conclusion:**

The competency-based, simulation-enhanced, and blended teaching model effectively improved learning outcomes and student engagement in the Fundamental Nursing Skills course. This reform model is feasible and has strong potential for wider application in higher vocational nursing education.

## 1 Introduction

The rapid transformation of healthcare systems, characterized by population aging, increasing disease complexity, and higher expectations for patient safety, has placed unprecedented demands on the professional competence of nursing personnel (1–4). As frontline healthcare providers, nurses are required not only to master solid theoretical knowledge but also to demonstrate proficient clinical skills, critical thinking, and effective communication abilities (5–8). Consequently, nursing education-particularly at the higher vocational level-must continuously adapt to ensure that graduates are adequately prepared for real-world clinical practice (9, 10).

Higher vocational nursing education places a strong emphasis on practical competence and job readiness (11, 12). Among the core foundational courses, Fundamental Nursing Skills plays a pivotal role in shaping students’ professional competence, as it provides essential training in basic nursing procedures, patient care techniques, and safety practices (13–16). This course serves as a critical bridge between classroom-based theoretical learning and subsequent clinical placements. The quality and effectiveness of teaching in this course directly influence students’ confidence, skill proficiency, and readiness for clinical environments (17–22).

However, traditional teaching approaches in fundamental nursing courses remain largely teacher-centered, relying heavily on lectures and instructor demonstrations (23). Although such approaches can efficiently transmit theoretical knowledge, they often limit students’ opportunities for active participation, hands-on practice, and reflective learning. Students may become passive recipients of information, resulting in fragmented skill acquisition and insufficient integration of theory with practice (24). These limitations are particularly problematic in vocational education, where the ultimate goal is to cultivate practice-oriented and clinically competent professionals.

In recent years, educational reform in nursing has increasingly emphasized competency-based education, which focuses on clearly defined learning outcomes aligned with clinical tasks and professional roles (25–29). Competency-based approaches shift the focus from knowledge transmission to the development of observable and measurable abilities, encouraging students to apply what they have learned in practical contexts. In parallel, simulation-based teaching has gained widespread recognition as an effective strategy for nursing education (30, 31). By recreating realistic clinical scenarios, simulation allows students to repeatedly practice nursing procedures in a safe and controlled environment, thereby reducing anxiety, enhancing confidence, and improving procedural accuracy (32).

Blended learning, which combines online and face-to-face instruction, has also emerged as an important pedagogical innovation. Through pre-class online learning, students can acquire foundational knowledge at their own pace, while in-class time can be devoted to skill practice, discussion, and feedback. This model not only improves learning efficiency but also supports individualized learning and active engagement—key elements for effective vocational education (33).

Despite the growing body of literature supporting competency-based, simulation-enhanced, and blended teaching strategies, empirical evidence regarding their integrated application in higher vocational nursing education remains limited. In particular, few studies have systematically evaluated the combined effects of these approaches on multiple learning outcomes within the Fundamental Nursing Skills course (34–36). Therefore, this study aimed to implement a comprehensive teaching reform model integrating competency-based curriculum design, simulation-based training, and blended learning, and to evaluate its effectiveness in improving theoretical knowledge, practical skills, learning engagement, and student satisfaction among higher vocational nursing students.

*Population*: higher vocational nursing students.

*Intervention*: competency-based, simulation-enhanced blended teaching mode.

*Comparison*: traditional lecture combined with skill demonstration teaching method.

*Outcomes*: theoretical knowledge level, practical operation skills, learning engagement and teaching satisfaction.

*Research question*: Does the application of competency-based and simulation-assisted blended teaching effectively promote theoretical mastery, practical ability and overall learning experience of higher vocational nursing students compared with traditional teaching methods?

## 2 Methods

### 2.1 Study design

A quasi-experimental study design was employed to evaluate the effectiveness of a teaching reform in the Fundamental Nursing Skills course (37). The intervention was implemented over one academic semester as part of routine teaching activities. The study aimed to compare learning outcomes between students receiving the reformed teaching model and those receiving traditional instruction.

A non-randomized quasi-experimental design was employed. Two intact parallel classes were selected; randomization was not feasible because students were assigned to classes according to the college’s fixed administrative arrangement.

### 2.2 Participants

Seventy-five full-time nursing students enrolled in a higher vocational college were recruited. Two parallel classes were assigned: one to the experimental group (*n* = 38) and the other to the control group (*n* = 37). Inclusion criteria were first-time enrollment in the Fundamental Nursing Skills course and no prior clinical nursing training. Exclusion criteria included withdrawal during the semester or incomplete assessment data (38–40).

Baseline characteristics, including age, gender, and prior academic performance, were collected. No significant differences were observed between groups (*p* > 0.05), confirming baseline equivalence (41). Beyond baseline comparisons of age, gender, and prior academic performance, key confounding variables were strictly controlled to ensure group equivalence. Both groups were taught by the same instructor following identical course content, teaching hours, materials, and assessment standards to eliminate instructor-related bias. Furthermore, all participants were first-year nursing students without prior clinical nursing exposure, which excluded the influence of previous clinical experience. The sample size was calculated via G*Power 3.1 software based on independent samples t-test. Setting the significance level *α* = 0.05 and statistical power = 0.80, combined with the medium effect size d = 0.60 referred to relevant nursing education studies, at least 34 participants were required in each group. Considering 10% possible participant dropout during the research period, we finally recruited 38 students in the experimental group and 37 students in the control group, with a total sample of 75 participants.

### 2.3 Teaching interventions

**Figure 1:**
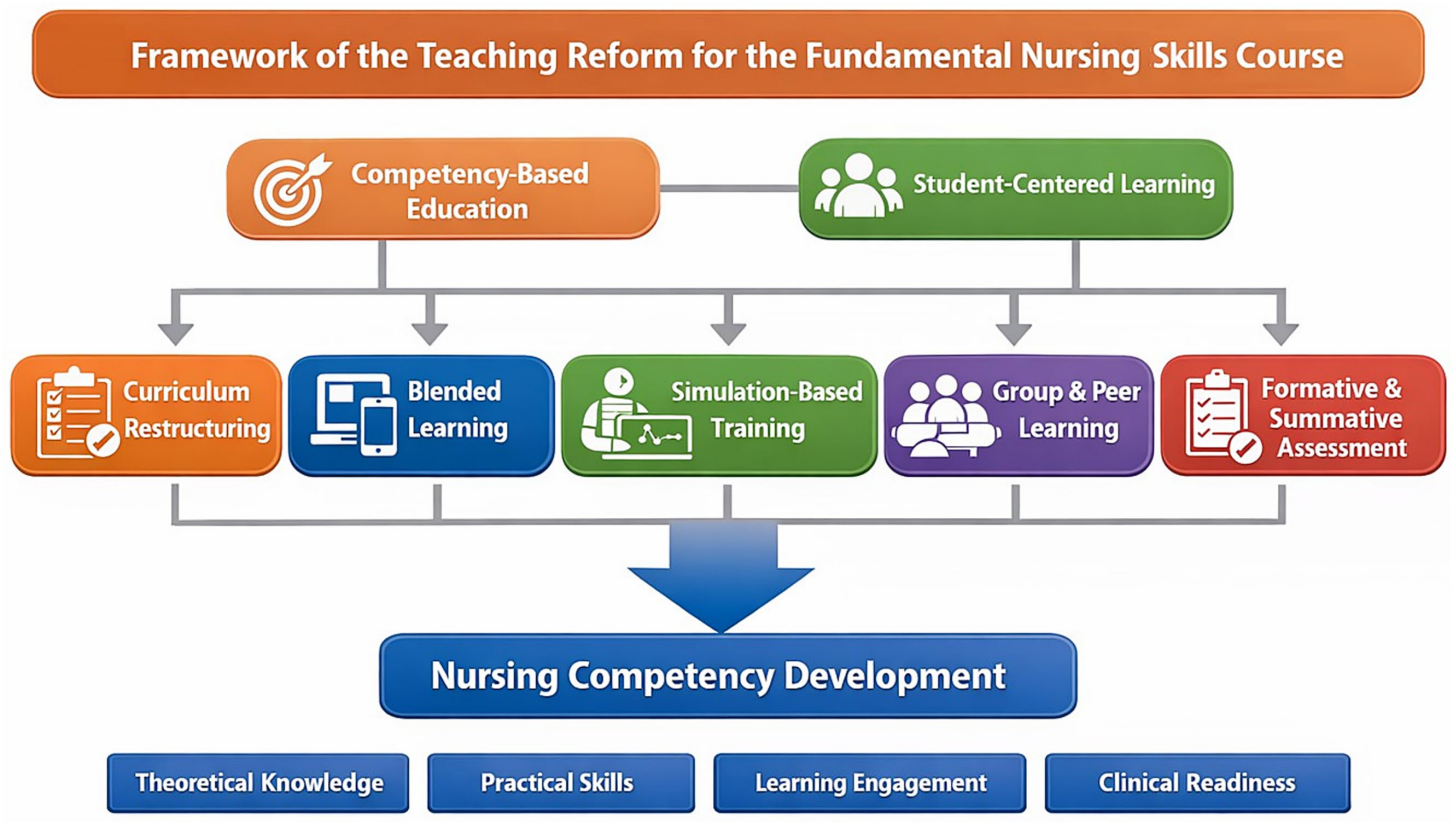
Conceptual framework of the competency-based teaching reform implemented in the Fundamental Nursing Skills course. The framework integrates curriculum restructuring, blended learning, simulation-based training, group-based learning, and formative–summative assessment to enhance nursing competencies.

**Figure 2:**
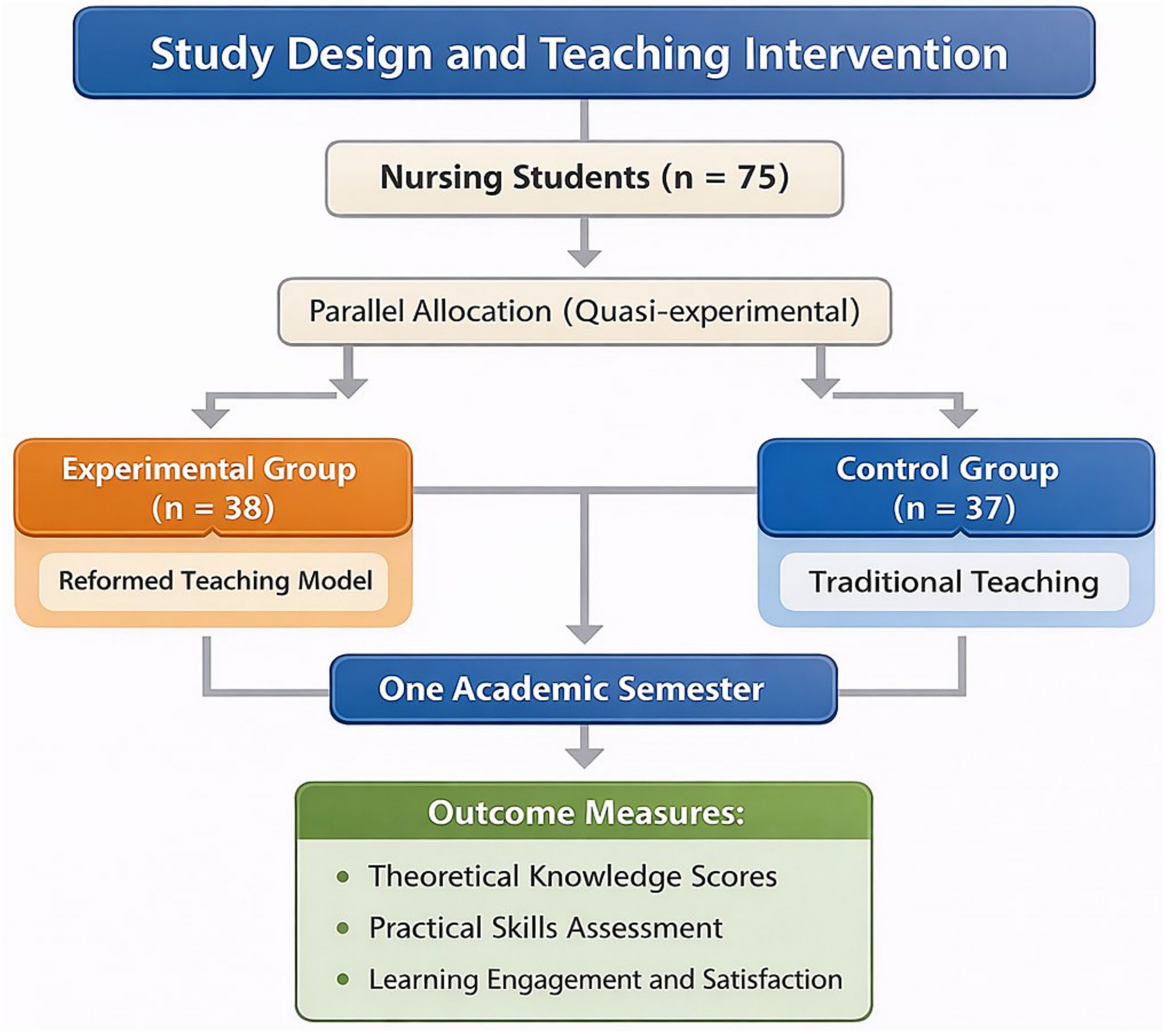
Flowchart illustrating the quasi-experimental study design, participant allocation, teaching interventions, and outcome assessment. Two parallel classes were assigned to either the experimental group (reformed teaching model) or the control group (traditional teaching), followed by evaluation of learning outcomes at the end of the semester.

The overall design of the competency-based teaching reform and the study workflow are illustrated in Figures 1, 2, respectively.

*Control group*: the control group received traditional teaching methods, consisting of lecture-based theoretical instruction, teacher-led demonstrations of nursing procedures, and a final summative skills examination at the end of the semester (42–44).

*Experimental group*: the experimental group received a reformed teaching model based on competency-oriented, student-centered, and practice-focused principles (45–48).

Key components included:

*Curriculum restructuring*: teaching modules were reorganized around core clinical competencies, emphasizing integration of theory and practice (49).

*Blended learning*: pre-class online learning resources, such as instructional videos and quizzes, were provided, while in-class time focused on hands-on practice and discussion (50–52).

*Simulation-based training*: mannequins and task trainers were used to enhance procedural skills, clinical reasoning, and safety awareness (53–55).

*Group-based learning and peer evaluation*: students worked in small groups to practice nursing skills, conduct peer assessments, and provide feedback (56, 57).

*Assessment integration*: formative assessments were conducted throughout the semester and combined with a final summative evaluation to comprehensively assess learning outcomes (58).

The intervention was implemented over one semester (18 weeks, 4 h per week). The weekly session structure comprised 1 h of online pre-class learning, 2 h of in-class lecture and demonstration, and 1 h of hands-on practice and simulation training. Simulation sessions were conducted twice weekly, 45 min per session, using medium-fidelity mannequins, with core scenarios including intravenous infusion, aseptic technique, and bed making. Competency-based modules focused on three key domains: (1) aseptic technique competency (e.g., sterile dressing change); (2) clinical skill competency (e.g., safe intravenous injection); and (3) patient safety competency (e.g., infection control and error prevention protocols).

### 2.4 Outcome measures

All outcome measures employed in this study were previously validated tools from published nursing education research and were not developed by the authors. At the end of the semester, the following four indicators were assessed.

Theoretical knowledge was evaluated using a standardized written examination covering core content of fundamental nursing skills (59–61).

Practical skills performance was assessed with a structured operational checklist that examined procedural accuracy, standardization, and safety awareness (59–61).

Learning engagement was measured using a validated Academic Engagement Questionnaire, which assesses vigor, dedication, and absorption (62). This instrument consists of 14 items (Vigor: 5 items, Dedication: 5 items, Absorption: 4 items) scored on a 5-point Likert scale from 0 (“never”) to 4 (“always”), yielding a total score between 0 and 56. A total score of ≥ 42 (mean item score ≥ 3) was predefined as the cutoff for “high learning engagement”, indicating that engagement behaviors occur “most of the time” or “always.” The Cronbach’s *α* of this scale in our study was 0.906 (62).

Student satisfaction was evaluated using the Nursing Student Satisfaction Scale (NSSS) (63). The NSSS is a 30-item, 6-point Likert-type measure (1 = “not satisfied” to 6 = “very satisfied”), covering three domains: Professional Social Interaction (9 items), Curriculum and Teaching (14 items), and Environment (7 items). The total score ranges from 30 to 180. A total score of ≥ 120 (mean item score ≥ 4) was predefined as the cutoff for a “satisfied” student. The Cronbach’s *α* coefficient for this scale was 0.93 (63).

### 2.5 Data collection and quality control

All assessments were conducted by trained instructors blinded to group allocation (64). Data were double-entered and cross-checked to ensure accuracy. Incomplete datasets were excluded from the final analysis (65).

### 2.6 Statistical analysis

Continuous variables are presented as mean ± standard deviation (SD). Normality was assessed using the Shapiro–Wilk test. Independent-samples *t* tests were used to compare continuous variables between groups, and chi-square (*χ*^2^) tests were applied for categorical variables, including learning engagement and student satisfaction (66, 67). All statistical tests were two-tailed, with *p* < 0.05 considered statistically significant. Analyses were performed using SPSS version 28.0 (IBM Corp., Armonk, NY, USA) (68).

## 3 Results

**Figure 3:**
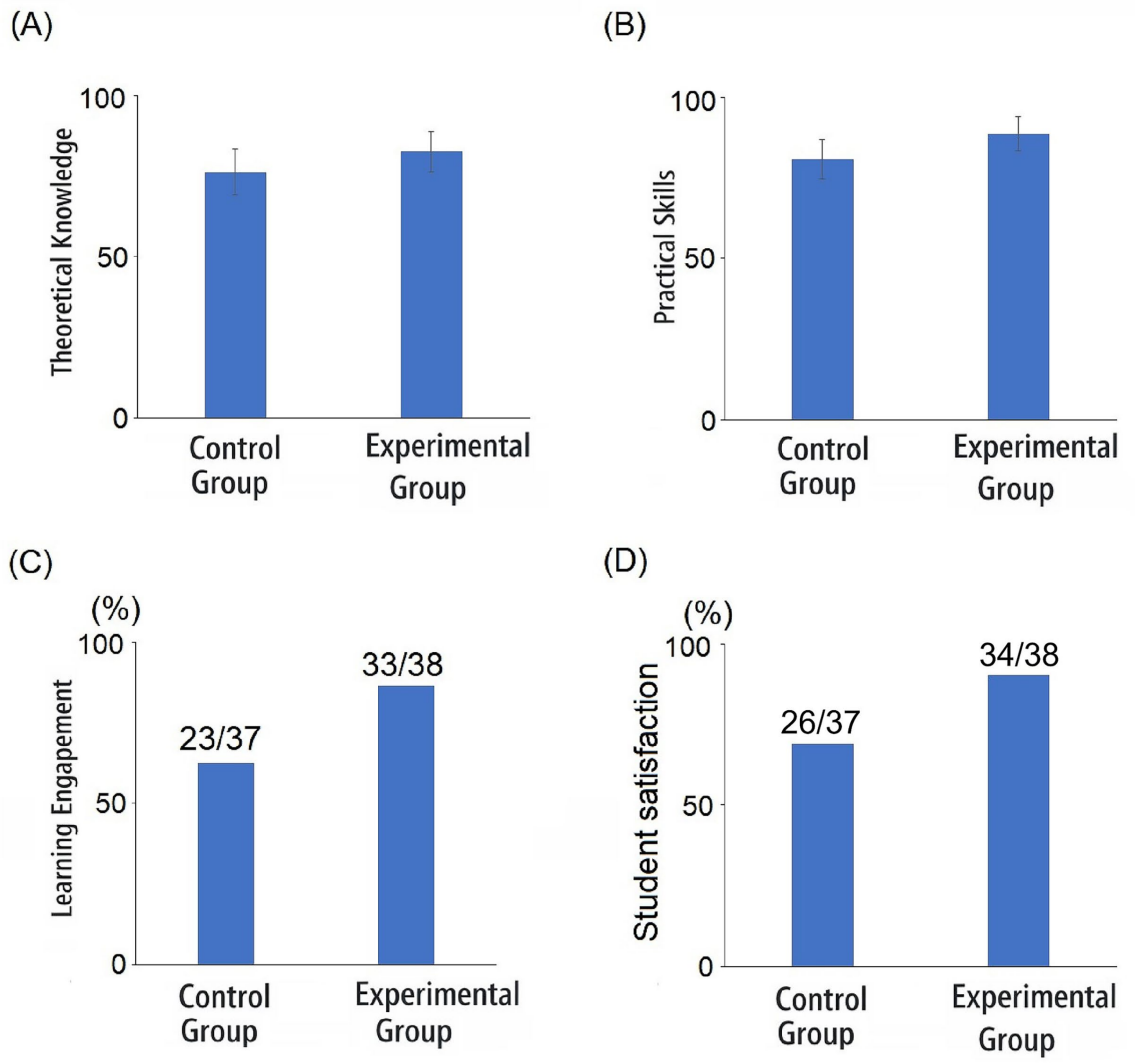
Comparison of learning outcomes between experimental and control groups. Bar chart illustrating differences in theoretical knowledge scores, practical skills scores, and student satisfaction between the experimental group and the control group at the end of the semester. Data are presented as mean ± standard deviation (SD). The experimental group demonstrated higher theoretical knowledge **(A)**, superior practical skills performance **(B)**, learning engagement **(C)**, and greater student satisfaction **(D)** compared with the control group, indicating an overall improvement in learning outcomes following the reformed teaching intervention.

The overall comparison of learning outcomes between the experimental and control groups is summarized in Figure 3 (69).

### 3.1 Comparison of theoretical knowledge scores

At the end of the semester, both groups completed a standardized theoretical examination on fundamental nursing knowledge. As shown in Figure 3, the experimental group achieved significantly higher theoretical knowledge scores than the control group.

The mean theoretical score of the experimental group was 82.47 ± 6.35, compared with 76.18 ± 7.02 in the control group. Independent-samples t-test analysis demonstrated a statistically significant difference between the two groups (t = 4.26, *p* < 0.001), indicating that the reformed teaching model effectively enhanced students’ knowledge acquisition (Table 1) (70, 71).

**Table 1:** Theoretical knowledge and practical skills scores.

Outcome	Experimental group (*n* = 38)	Control group (*n* = 37)	*t*/*χ*^2^	*p*
Theoretical knowledge (mean ± SD)	82.47 ± 6.35	76.18 ± 7.02	4.26	<0.001
Practical skills (mean ± SD)	88.63 ± 5.41	80.72 ± 6.18	6.01	<0.001

SD, standard deviation; *t*, independent-samples *t*-test.

### 3.2 Comparison of practical skills performance

Practical nursing skills were evaluated using a structured operational assessment checklist covering procedural standardization, operational accuracy, safety awareness, and overall performance (72).

The experimental group demonstrated significantly better practical skills performance, with a mean score of 88.63 ± 5.41, compared with 80.72 ± 6.18 in the control group (*t* = 6.01, *p* < 0.001). Improvements were particularly evident in adherence to standard operating procedures, correct use of nursing instruments, and effective patient communication during simulated clinical scenarios.

### 3.3 Learning engagement and classroom participation

Learning engagement was assessed through classroom participation records and a validated questionnaire (62). The experimental group demonstrated significantly higher levels of active participation during classroom activities and practical training sessions.

Questionnaire results indicated that, using the predefined cutoff (total score ≥ 42), 86.8% (33/38) of students in the experimental group were classified as having high or very high learning engagement, compared with 62.2% (23/37) in the control group (*χ*^2^ = 6.04, *p* = 0.014). Students in the experimental group were more willing to participate in group discussions, peer evaluations, and hands-on practice, reflecting enhanced learning motivation and initiative.

### 3.4 Student satisfaction with teaching methods

Student satisfaction with teaching methods was evaluated using a standardized questionnaire covering teaching content, instructional methods, classroom atmosphere, and perceived learning effectiveness (63).

Using the predefined cutoff (total score ≥ 120), 89.5% (34/38) of students in the experimental group were classified as satisfied with the teaching methods, compared with 70.3% (26/37) in the control group (*χ*^2^ = 4.32, *p* = 0.038). Students expressed strong approval of the integration of online and offline learning, simulation-based training, and diversified assessment strategies.

### 3.5 Summary of teaching reform outcomes

Taken together, the results demonstrate that the reformed teaching model significantly outperformed the traditional approach across multiple outcome measures, including theoretical knowledge, practical skills, learning engagement, and student satisfaction. These findings provide robust empirical evidence supporting the effectiveness of competency-based, simulation-enhanced, and blended teaching reform in the Fundamental Nursing Skills course (34–36, 45–48).

## 4 Discussion

This study provides empirical evidence that a competency-based, simulation-enhanced, and blended teaching model can significantly improve learning outcomes in the Fundamental Nursing Skills course within higher vocational nursing education (34–36, 45–48). Unlike traditional teaching approaches that emphasize passive knowledge acquisition, the reformed model promotes active learning and practical competence development, which are central goals of vocational nursing education (23, 24).

The improvement in theoretical knowledge observed in the experimental group may be attributed to the alignment of teaching content with clearly defined competencies and clinical tasks (25–29). By restructuring the curriculum around practical nursing competencies, students were better able to understand the relevance of theoretical concepts and apply them in practice-oriented learning activities. Blended learning further supported this process by allowing students to acquire foundational knowledge through online resources prior to class, thereby freeing classroom time for clarification, discussion, and application (33, 50–52). This pedagogical shift likely facilitated deeper cognitive processing and improved knowledge retention.

Simulation-based training played a particularly important role in enhancing students’ practical skills performance (30–32, 53–55). Through repeated exposure to simulated clinical scenarios, students could practice nursing procedures in a safe environment, receive immediate feedback, and correct errors without the risk of harming patients. Such experiential learning opportunities are difficult to achieve through traditional demonstration-based teaching alone. Simulation may also help reduce performance anxiety and enhance self-efficacy, enabling students to perform nursing procedures more confidently and accurately (31, 32).

The significant increase in learning engagement and student satisfaction observed in the experimental group suggests that the reformed teaching model successfully created a more student-centered and motivating learning environment (56, 57, 62, 63). Group-based learning and peer evaluation encouraged active participation and collaboration, allowing students to learn from both instructors and peers. These interactive learning experiences may foster a sense of responsibility, autonomy, and professional identity, which are essential for sustaining motivation in vocational education settings.

From an educational perspective, the findings of this study support the notion that effective nursing education should move beyond knowledge transmission and focus on the holistic development of competencies (1–4, 9–12). Curriculum designers and educators in higher vocational nursing programs should consider integrating competency-based frameworks, simulation-based training, and blended learning strategies to enhance teaching effectiveness. In addition, assessment systems should incorporate formative evaluation throughout the learning process, rather than relying solely on final examinations, to better reflect students’ ongoing development (58).

Consistent with Du et al. (21), who systematically verified the advantages of blended teaching in nursing education, our study also found that competency-based blended teaching significantly improved students’ theoretical knowledge and practical skills compared with traditional teaching. Similarly, a meta-analysis by Kim et al. (73) confirmed that simulation-based education significantly enhances nursing students’ clinical skills and active participation, with higher-fidelity simulations producing stronger effects. In contrast, a randomized controlled trial by Ecoff et al. (74) found no significant differences in nurses’ knowledge scores between pre-test and post-test across different educational methods. Such inconsistent results may be attributed to the absence of systematic competency-based training and insufficient integration of simulation-enhanced learning, whereas the superior effects in our study are likely due to the 18-week continuous teaching design, standardized simulation sessions, and complete competency-oriented teaching system, which progressively consolidated students’ knowledge and skills and maintained long-term learning motivation.

The significant improvements observed in the experimental group can be explained by constructivist learning theory and experiential learning theory. Constructivism emphasizes that learners actively build knowledge through practice and reflection, which aligns with our student-centered, competency-based design. Experiential learning theory further supports our results: repeated simulation practice allowed students to engage in concrete experience, reflective observation, abstract conceptualization, and active experimentation, strengthening skill retention and clinical reasoning. Notably, Sherwood and Francis (75) reported in a systematic review that the advantages of simulation-based training did not persist beyond the immediate post-intervention period, with insufficient evidence at 1–3 weeks follow-up. The stronger effects in our study likely stem from the 18-week comprehensive model integrating structured online pre-learning, systematic simulation training, and ongoing formative assessment—elements that sustained student motivation and aligned learning closely with real clinical demands.

Several limitations of this study should be acknowledged. First, the sample size (*n* = 75) is small and recruited from a single vocational college, which may limit external validity and generalizability. Future multi-center studies with larger samples are warranted (37, 38). Second, the study focused on short-term learning outcomes assessed at the end of the semester, and did not evaluate long-term retention of knowledge or clinical performance during internships. Future research should involve multi-center studies with larger samples and longitudinal designs to examine the sustained impact of teaching reforms on students’ clinical competence and professional development (35, 36).

In conclusion, the present study demonstrates that a competency-based, simulation-enhanced, and blended teaching model is effective in improving theoretical knowledge, practical skills, learning engagement, and student satisfaction in the Fundamental Nursing Skills course. This teaching reform provides a feasible and evidence-based approach for optimizing foundational nursing education in higher vocational settings. Wider implementation of such teaching models may contribute to the cultivation of practice-oriented, competent, and confident nursing professionals who are better prepared to meet the demands of modern healthcare systems.

## Data Availability

The raw data supporting the conclusions of this article will be made available by the authors, without undue reservation.
